# Insights on the evolution of the living Great Amazon Reef System, equatorial West Atlantic

**DOI:** 10.1038/s41598-019-50245-6

**Published:** 2019-09-23

**Authors:** Michel Michaelovitch de Mahiques, Eduardo Siegle, Ronaldo Bastos Francini-Filho, Fabiano Lopes Thompson, Carlos Eduardo de Rezende, José Diego Gomes, Nils Edvin Asp

**Affiliations:** 1 Oceanographic Institute of the University of São Paulo, São Paulo, Brazil; 2 Institute of Energy and Environment of the University of São Paulo, São Paulo, Brazil; 3 Federal University of Paraíba, Paraíba, Brazil; 4 Federal University of Rio de Janeiro, Rio de Janeiro, Brazil; 5 State University of Norte Fluminense, Rio de Janeiro, Brazil; 6 Federal University of Pará, Belém, Brazil

**Keywords:** Stratigraphy, Sedimentology, Marine biology

## Abstract

The Great Amazon Reef (GARS) is an extensive mesophotic reef ecosystem between Brazil and the Caribbean. Despite being considered as one of the most important mesophotic reef ecosystems of the South Atlantic, recent criticism on the existence of a living reef in the Amazon River mouth was raised by some scientists and politicians. The region is coveted for large-scale projects for oil and gas exploration. Here, we add to the increasing knowledge about the GARS by exploring evolutionary aspects of the reef using primary and secondary information on radiocarbon dating from carbonate samples. The results obtained demonstrate that the reef is alive and growing, with living organisms inhabiting the GARS in its totality. Additional studies on net reef growth, habitat diversity, and associated biodiversity are urgently needed to help reconcile economic activities and biodiversity conservation.

## Introduction

**Figure 1:**
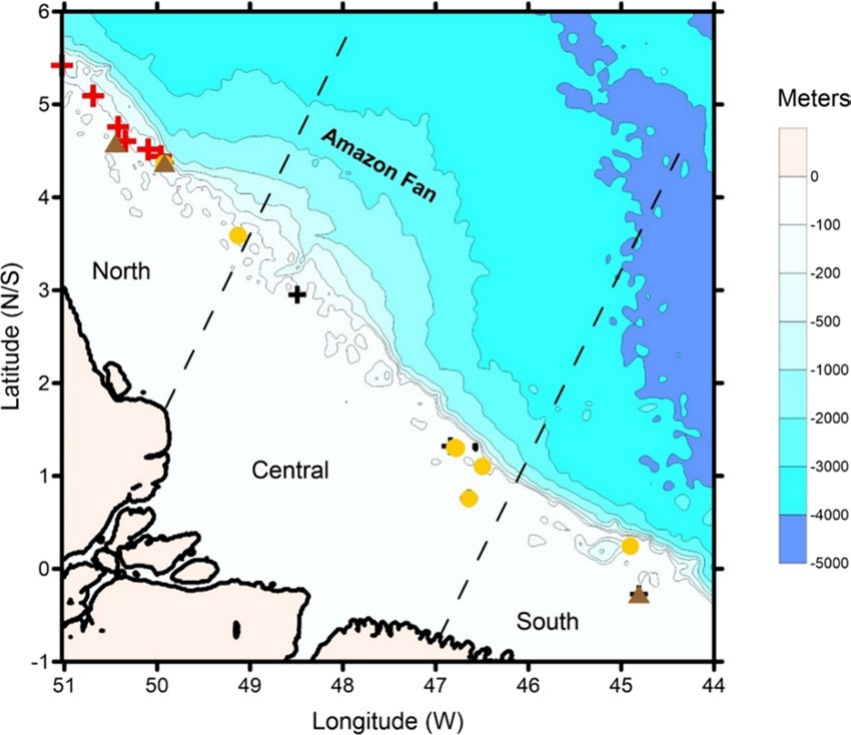
Location of the study area, sectors as defined by Moura et al. (2016), and samples analysed in this study. Source of information for samples: Red cross^8^, Brown triangle^3^, Black cross^37^, Yellow dot (this paper).

Although described 40 years ago by^1^, the Great Amazon Reef (GARS; northern limit of the Brazilian Province) was only recently recognized as an extensive and diverse reef system in the Amazon continental margin, between Brazil and the Caribbean^2–5^ (Fig. 1).

The GARS is currently considered as one of the most important mesophotic reef ecosystems of the South Atlantic^6^. It is composed by a mosaic of shallow patch reefs (50–70 m), mesophotic reefs (30–220 m depth), large living rhodolith beds and more complex living hard bottoms (formed mainly by fused calcareous algae) inhabited by a diverse reef biota, including threatened and commercially important species^4^. Biogenic reefs (patch reefs, platforms and walls) are mostly composed by crustose calcareous algae, with sparse areas covered by scleractinian corals, particularly *Madracis decactis*^4^.

**Figure 2:**
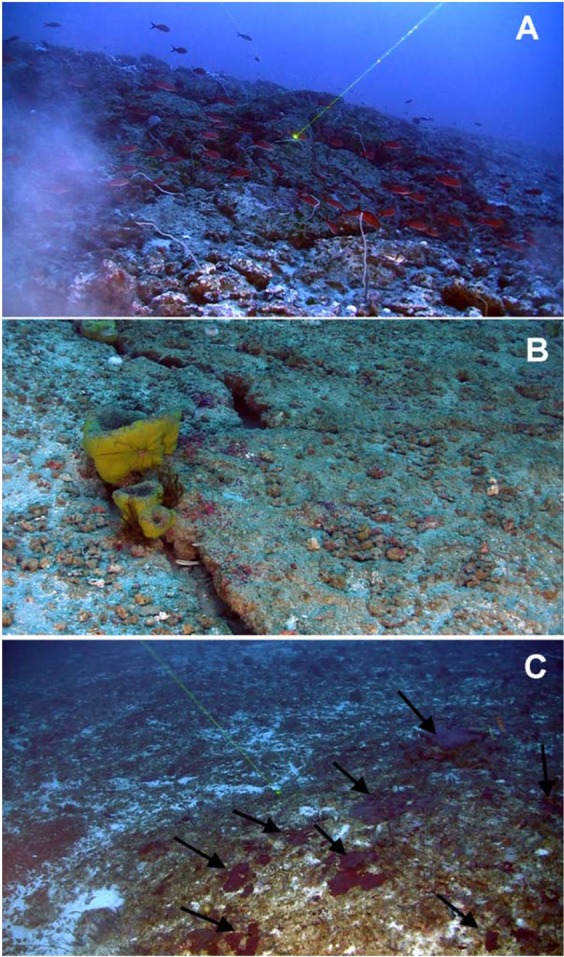
(**A**) a complex reef emerging >4 m in height from the bottom formed by living crustose coralline algae and covered with black corals (elongated white structures). (**B**) Fractures evidencing carbonatic platforms sparsely covered by rhodoliths and sponges and (**C**) a patch reef covered with the scleractinian coral *Madracis decactis* (black arrows). Photos taken by R.B.F.F.

Despite its plausible socio-ecological importance, the GARS is highly threatened by the expansion of oil and gas exploration projects in the region. In 2018, the existence of the GARS itself started to be questioned by a few scientists^7^, as well as by industrial sectors and politicians in favour of oil exploration in the region. The main argument is that there are no large biogenic structures built by living corals and/or other reef-building organisms (e.g., calcareous algae), particularly in the northern portion of the GARS, due to larger influx of sediments from the Amazon River across the continental shelf^7^, which may have led to a relict reef environment, developed during MIS2^8^. Because of such scepticism, a more detailed description of the GARS was provided by^4^. These authors showed that the GARS is composed of large and extensive biogenic structures build mainly by living calcareous algae (“coralline algal frameworks,” cf^9^.) (Fig. 2). More recently^10^, have demonstrated that there is enough light for photosynthetic organisms to thrive in the area of the GARS.

The older dating ages used in^7^ were published by^8^ and would correspond to the oolites present in the Northern Sector. However^11^, argued that these oolite samples were displaced from their original place of formation and highlighted one should be very careful when using these dated oolite samples to infer reef age and accretion^7^. also stated that the Amazon shelf is mostly covered by muds, a condition which would impede the development of carbonate reefs. Indeed, the inner and middle parts of the Amazon shelf are mostly covered by muds^12,13^, while the outer shelf is covered by coarser sediments and consolidated substrates, as reported by^13^ and confirmed by the images reported by^4^.

The predominance of terrigenous sediments, particularly in the inner and mid-shelf portions of the Amazonian shelf, is not a limiting factor for the existence of biogenic reefs, as there is enough light available for photosynthetic organisms across most of the Amazon shelf, even in the north sector^10^. Another example of turbid-water reefs include the most extensive coral reefs in the SW Atlantic (Abrolhos Bank, central Brazilian coast), which are surrounded mostly by siliciclastic sediments^14,15^. Brazilian biogenic reef ecosystems are recognised for being well adapted to high turbidity levels due to terrestrial input (via large rivers discharge) and sediment resuspension^14,16^.

The GARS is located mostly at the middle, and outer shelf of the Foz do Amazonas and Pará-Maranhão marginal sedimentary basins within a depth range of 70 to 220 m^4^. The area is substantially influenced by the Amazon River, with a mean annual water discharge on the order of 6.0 10^12^ m^3^ and annual suspended-sediment load on the order of 1.2 10^9^ tons^17^, affecting all physical and chemical aspects of the water column (e.g., salinity, light availability, pH, dissolved nutrients) over the shelf including the GARS. Most of the sediment travels north in a plume driven by the northwest-flowing North Brazil and Guiana Currents, which are forced by local wind patterns^18^.

The Amazon River plume has a very dynamic character and even though it is generally driven to the northwest, seasonal variations of winds and currents on the shelf lead to some southeastward expansion of the plume, as a result of displacements caused by the Inter-Tropical Convergence Zone (ITCZ), with substantial rainfall and NE Trade Winds between January and June. In contrast, between July and December rainfall is reduced, and SE Trade Winds prevail^19^. There are four distinct zones based on light regimes reaching the bottom, three of them with constant light regimes (dark coastal zone under the permanent influence of the plume; dim-light zone in the deeper northern shelf and high-light zone in the shallower southern shelf) and one zone with seasonal changes in benthic light regimes (northern mid-to-outer shelf)^10^.

Waves reach the region mainly from the east and northeast, because of the NE-SE trade winds, with dominant offshore wave heights between 1 and 3 m. The most energetic waves approach the region between December and March, with strong NE Trade Winds offshore^20^. Besides the oceanic currents and waves, tidal water level variation and tidal currents are also substantial in the area, with tidal amplification on the shelf resulting in macrotidal conditions at the coast^21^.

In this paper, we aim to summarise published and unpublished radiocarbon dating from carbonate samples from the GARS in order to: 1) evaluate its modern or relict nature and 2) contribute to the understanding of its evolution by proposing a theoretical model from the end of Marine Isotope Stage 2 until modern ages.

## Results

Table 1 summarizes the radiocarbon ages presented in this work.

**Table 1:** Summary of the radiocarbon data used in this work.

Sample	Sector	Latitude (N/S)	Longitude (W)	Depth (m)	Lab Number	Material	Conventional Age (BP)	Calibrated Age (Median Probability) BP	95% range (cal BP)	Fraction Modern Carbon	D14C (‰)	Reference
V-18-18-Top	North	5.420	−51.027	150		Oolite	20090 ± 600	23710	22405–25205	Not available	Not available	Milliman & Barretto (1975)
V-18-18-Bottom	North	5.420	−51.027	150		Oolite	38000	41864	40020–43350	Not available	Not available	Milliman & Barretto (1975)
X-236	North	5.091	−50.690	133		Oolite	17010 ± 400	20037	19025–20995	Not available	Not available	Milliman & Barretto (1975)
G209	North	4.757	−50.419	109		Oolite	14470 ± 400	17045	15950–18060	Not available	Not available	Milliman & Barretto (1975)
G210	North	4.604	−50.343	104		Oolite	14310 ± 250	16845	16150–17545	Not available	Not available	Milliman & Barretto (1975)
1921	North	4.513	−50.097	146		Oolite	17170 ± 1240	20276	17350–23265	Not available	Not available	Milliman & Barretto (1975)
G211	North	4.440	−49.960	191		Oolite	21250 ± 400	25063	24145–25885	Not available	Not available	Milliman & Barretto (1975)
N01	North	4.370	−49.920	120		Carbonate	12620 ± 30	14105	13975–14225	Not available	Not available	Moura *et al*.^3^
C01-Surface	North	4.580	−50.450	80		Carbonate	4480 ± 25	4680	4570–4790	Not available	Not available	Moura *et al*.^3^
C02-Core	North	4.580	−50.450	80		Carbonate	6950 ± 30	7115	7005–7210	Not available	Not available	Moura *et al*.^3^
S01	South	−0.270	−44.810	23		Carbonate	Modern	Modern		Not available	Not available	Moura *et al*.^3^
N01	North	4.370	−49.920	120		Oolite		12100	Not available	Not available	Not available	Vale *et al*.^37^
N01	North	4.370	−49.920	120		Bivalve shell		13480	Not available	Not available	Not available	Vale *et al*.^37^
N12	North	4.370	−49.920	120		Coral polyp		14680	Not available	Not available	Not available	Vale *et al*.^37^
R14	North	2.950	−48.490	95		Bryozoan		2460	Not available	Not available	Not available	Vale *et al*.^37^
R14	North	2.950	−48.490	95		CCA		2050	Not available	Not available	Not available	Vale *et al*.^37^
R17	Central	1.320	−46.840	55		Bryozoan		680	Not available	Not available	Not available	Vale *et al*.^37^
R17	Central	1.320	−46.840	55		Hydrocoral		560	Not available	Not available	Not available	Vale *et al*.^37^
R07	Central	0.760	−46.640	50		CCA		1300	Not available	Not available	Not available	Vale *et al*.^37^
R07	Central	0.760	−46.640	50		CCA		510	Not available	Not available	Not available	Vale *et al*.^37^
R07	Central	0.760	−46.640	50		CCA		1040	Not available	Not available	Not available	Vale *et al*.^37^
R07	Central	0.760	−46.640	50		CCA		580	Not available	Not available	Not available	Vale *et al*.^37^
N18	South	−0.270	−44.810	23		Hydrocoral	Modern	Modern		Not available	Not available	Vale *et al*.^37^
NB3/1 FT59	North	3.590	−49.127	95	Beta-516488	Sponge	Modern	Modern		1.0342 ± 0.0039	34.18 ± 3.86	This work
NB3/1 FT52	North	3.590	−49.127	95	Beta-516487	Rhodolith	Modern	Modern		1.0188 ± 0.0038	18.85 ± 3.81	This work
NB2/1 FT55	North	4.368	−49.926	120	Beta-524179	CCA	Modern	Modern		1.0113 ± 0.0038	11.27 ± 3.78	This work
NB3/1 FT52	North	3.590	−49.127	95	Beta-524180	Rhodolith	50 ± 30	Modern		0.9938 ± 0.0037	−6.21 ± 3.71	This work
NB6/1 UFRJ14	Central	1.300	−46.779	55	Beta-524181	Rhodolith	Modern	Modern		1.0406 ± 0.0039	40.64 ± 3.89	This work
NB6/1 UFRJ13/FT31	Central	1.300	−46.779	55	Beta-524182	Rhodolith	Modern	Modern		1.0290 ± 0.0038	29.05 ± 3.84	This work
NB6/1 FT14	South	1.305	−46.797	60	Beta-516486	Sponge	940 ± 30	540	490–610	0.8896 ± 0.0033	−110.43 ± 3.32	This work
NB7/1 FT29	South	1.103	−46.495	100	Beta-516490	Rhodolith	170 ± 30	Modern		0.9791 ± 0.0037	−20.94 ± 3.66	This work
NB8/1 FT50	South	0.756	−46.642	40	Beta-524183	Rhodolith	Modern	Modern		1.0227 ± 0.0038	22.66 ± 3.82	This work
NB10/2 FT08	South	0.246	−44.901	23	Beta-524184	Rhodolith	270 ± 30	Modern		0.9669 ± 0.0036	−33.05 ± 3.61	This work

**Figure 3:**
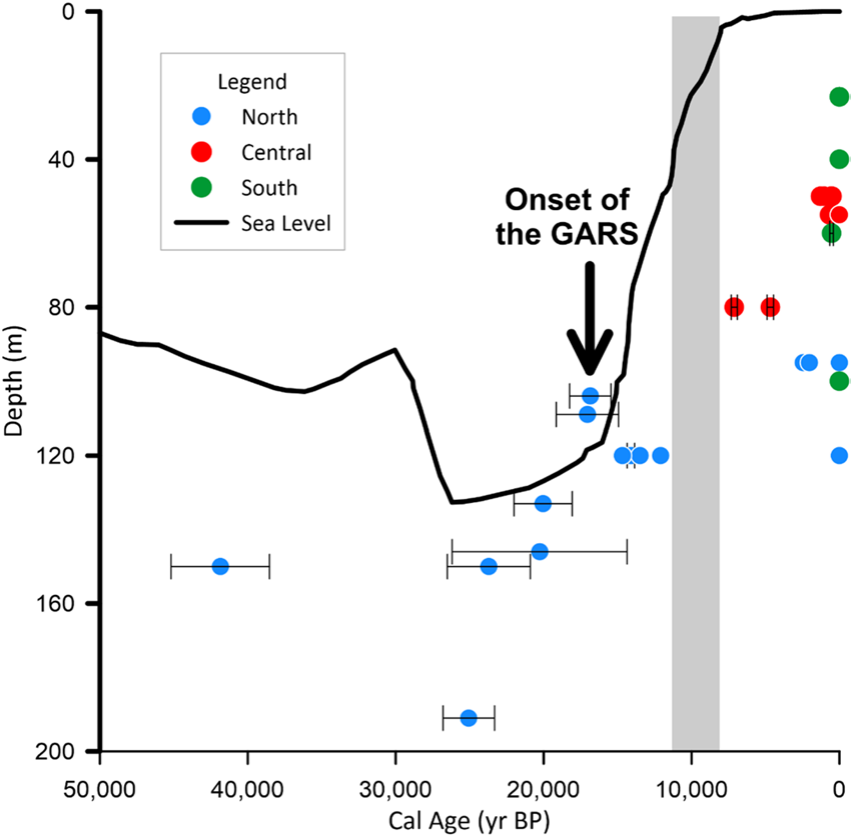
Bathymetric distribution of the samples dated, associated with a general sea-level curve^25^. The possible age of the onset of the GARS, based in our revision, is shown with a black arrow. The grey area shows the time gap, referred to on the Discussion.

Radiocarbon ages can be divided into three main groups (Fig. 3):

i. Contains samples exclusively from the northern sector, at water depths between 104 and 150 meters, encompassing samples with ages from the MIS2, as defined by^22^, with three exceptions, the first corresponding to an age of ca. 41,800 cal BP, and two others, with ages corresponding to the Younger Dryas, as delimited by^23^. The oldest ages correspond to the dating of oolites, published by^8^;
ii. Encompasses samples from the North and Central sectors, and presents a period from ca. 7,100 cal BP (Mid-Holocene), to historical ages (non-modern radiocarbon ages). Water depth from these samples varies from 50 to 95 meters, and;
iii. Corresponds to samples with modern radiocarbon ages, as defined by^24^ and contains samples from the three sectors. The water depth range of these samples varies from 23 to 100 meters; the shallowest samples are located in the Southern sector.

## Discussion

**Figure 4:**
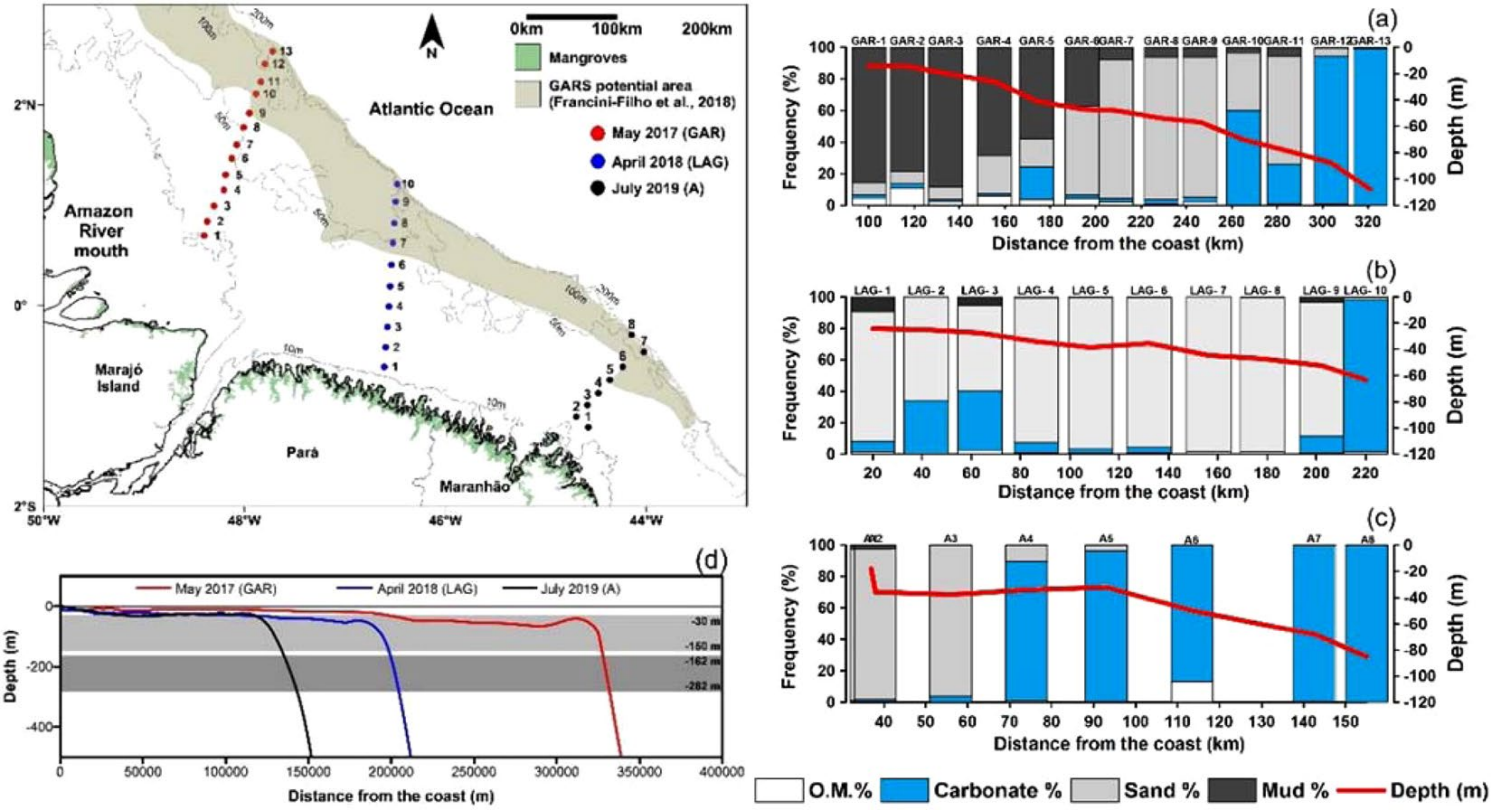
Bottom sedimentary characteristics and morphology of three profiles at the Amazon shelf stressing the carbonatic-siliciclastic sedimentation contrast (**a–c**) and the mesophotic depth range in contrast to the bottom morphology and present (light grey) and LGM (deep grey) sea level (**d**).

Cross-shelf profiles of sediment samples obtained between 2017 and 2019 show that the morphology and the sedimentary characteristics from the central and southern sectors of the GARS support the ages compiled here (Fig. 4). Considering the mesophotic depth range of 30 to 150 m, local depths and the sea level variation^25^, the area of the potential occurrence of mesophotic reefs during the Last Glacial Maximum (LGM) was rather narrow and restricted to the continental slope. As sea level rose quickly from LGM towards Mid-Holocene, there was a period of restriction of areas within the mesophotic depth range, before the drowning of the shelf.

The bathymetric profile from the central sector shows a substantially wider shelf, within a deeper outer shelf (Fig. 4), favouring reef development during Mid-Holocene. On the other hand, the distance from the Amazon River mouth and the shallowness of the shelf at the south favoured relatively shallow reef development there, with shallow reefs expanding their occurrence towards the central sector. A recent study focusing on fisheries in the Amazon River mouth suggests that reef structures may occur in areas that are much shallower than previously anticipated^26^.

A recent review of the formation of oolites^27^ confirms the intertidal and shallow subtidal formation of most of the marine oozes but emphasizes the complexity of the biogeochemical processes involved in their formation. When comparing the position of the oolites dated by^8^ with the sea-level change curve (Fig. 3), we agree with the observations by^11^ about the lack of reliability of oolite materials as indicators of ancient shores on the Amazon margin.

In this sense, we state that the MIS2 and MIS 3 ages, presented by^8^ and used by^7^ as for the onset of the GARS cannot be used for an evolutionary model for the reef system. Assuming that the oldest ages must be analysed with care, the first reliable dating to be considered for the GARS correspond to the samples, located on the Northern sector, that lie between 14,680 and 12,100 cal BP, at a water depth of 120 meters, in synchronicity with the Heinrich H1^28^.

Considering these ages and the sea-level curve shown in Fig. 2^25^, we propose a model of the evolution of GARS throughout the Late Quaternary, comprising three major phases:

i. We could assume that a first phase of the development of the reef occurred at water depths between 50 and 100 meters, approximately, right in the central range of mesophotic reef ecosystems^29^. This phase would occur at the end of the Pleistocene, between ca 14,700 and 12,100 cal BP;
ii. After the first phase, there is a gap in radiocarbon ages, corresponding to the interval between 12,100 and 7,100 cal BP, followed by the beginning of the occurrence of reef material in the Central sector. This gap roughly corresponds in time to the acceleration of the sea-level rise after the Younger Dryas (Melt Water Pulse 1B)^30,31^. Further, the time of the gap also follows the period when the Amazon River sediment load was developing a prominent plume, whereas during the previous period the sea-level was close to the shelf break and most of the Amazon sediment was transported into the deep sea by way of the Amazon Submarine Canyon and smaller channels^32^. Within the strong plume development, light penetration in the water column would be strongly attenuated, impairing the reef development. This process attenuated as the sea-level rise went on and the Amazon River plume moved landward and northwestwards.
iii. Finally, modern ages are found in samples from the three sectors, indicating the spread of the reef complex, from Northwest to Southeast. Also, reef constituents are found in areas as shallow as 20 meters (in the southern sector), and as deep as 220 meters^4^.

Our radiocarbon dating study is coherent with previous studies performed on rhodoliths from the Pacific and the Atlantic oceans where metagenomics demonstrated that the majority of living material corresponds to microbes (bacteria) capable of growing in a variety of extreme environmental conditions for carbonate precipitation^33,34^. Also, it was clear from these previous studies, that rhodoliths have a fraction of living carbonate cryptofauna and/or epifauna, including Foraminifera (*Homotrema rubrum*), Polychaeta (encrusting calcareous tubes), Bryozoa, and Mollusca (Vermetidae) phyla^33^.

The GARS is considered an ecotone of biodiversity between Brazil and the southern Caribbean, possibly acting as an ecological corridor between the South and North Atlantic. It comprises a high diversity of habitats and large areas dominated by healthy reef-building organisms (mostly crustose calcareous algae)^4^. Reef growth and high structural complexity are presently concentrated in the central and southern sectors, which are shallower and with higher light incidence over the bottom wealth. Net reef growth estimates (i.e. considering both, reef accretion and erosion) are scarce even for shallow reefs. Only recently, this question started to be addressed for mesophotic reefs^35,36^. Similar studies are urgently needed for the GARS, particularly considering its ecological importance and the threats posed by large scale oil and gas exploration in the region^4^.

## Conclusions

The analysis of previously published and new radiocarbon data from the GARS allowed us to recognize a Northwest-Southeast growing trend of the reef complex. Older ages correspond to samples from the northern sector, in areas located below the present plume of the Amazon River. These ages refer to the carbonates that sustained the reef during MIS2 and to the beginning of the last deglacial. After a gap, occurred between ca. 12,100 and 7,100 cal BP, the reef extended to the Central and Southern sector of the area. This gap corresponds to the time interval of acceleration of sea-level rise and plume development, after the Younger Dryas (Melt Water Pulse 1B).

Modern radiocarbon ages, obtained in rhodoliths and sponges, are present in the three sectors, indicating that differently than suggested, living organisms inhabit the GARS in its totality.

## Methods

As a convention, in this work, we keep the same proposal of three sectors (North, Central, and South) as stated by^3^. Besides our new radiocarbon data, we gathered dating information from other sources^3,8,37^ in order to evaluate the modern or relict character of the reef complex. When available, all of the conventional radiocarbon datings were recalibrated using software Calib 7.1^38^, using the Marine13 calibration curve^39^ and a global reservoir effect. Ages published in^37^ are presented “as is” (only calibrated ages, without the 2σ interval), since we did not have access to the original data. We also dated ten other samples (Table 1) of carbonates from different marine organisms from the three sectors at Beta Analytic (Miami, USA).

In order to compare our data with sea-level changes, and due to a lack of a reliable Late Quaternary sea-level curve for northern Brazil, we used the global curve proposed by^22^.

The location of the samples is shown in Table 1 and Fig. 1.
